# Health care utilization and outpatient, out-of-pocket costs for active convulsive epilepsy in rural northeastern South Africa: a cross-sectional Survey

**DOI:** 10.1186/s12913-016-1460-0

**Published:** 2016-06-28

**Authors:** Ryan G. Wagner, Melanie Y. Bertram, F. Xavier Gómez-Olivé, Stephen M. Tollman, Lars Lindholm, Charles R. Newton, Karen J. Hofman

**Affiliations:** Studies of Epidemiology of Epilepsy in Demographic Surveillance Systems (SEEDS) – INDEPTH Network, Accra, Ghana; MRC/Wits Rural Public Health & Health Transitions Research Unit (Agincourt), School of Public Health, Faculty of Health Sciences, University of the Witwatersrand, Johannesburg, South Africa; Epidemiology and Public Health Sciences, Department of Public Health and Clinical Medicine, Umeå University, Umeå, Sweden; World Health Organization, Geneva, Switzerland; International Network for the Demographic Evaluation of People and their Health (INDEPTH) Network, Accra, Ghana; KEMRI/Wellcome Trust Research Programme, Centre for Geographic Medicine Research – Coast, Kilifi, Kenya; Neurosciences Unit, UCL Institute of Child Health, London, United Kingdom; Clinical Research Unit, London School of Hygiene and Tropical Medicine, London, United Kingdom; Department of Psychiatry, University of Oxford, Oxford, United Kingdom; Priority Cost-effective Lessons for Systems Strengthening (PRICELESS), Johannesburg, South Africa

**Keywords:** Africa, Direct medical costs, Indirect medical costs, Population-based, Chronic disease

## Abstract

**Background:**

Epilepsy is a common neurological disorder, with over 80 % of cases found in low- and middle-income countries (LMICs). Studies from high-income countries find a significant economic burden associated with epilepsy, yet few studies from LMICs, where out-of-pocket costs for general healthcare can be substantial, have assessed out-of-pocket costs and health care utilization for outpatient epilepsy care.

**Methods:**

Within an established health and socio-demographic surveillance system in rural South Africa, a questionnaire to assess self-reported health care utilization and time spent traveling to and waiting to be seen at health facilities was administered to 250 individuals, previously diagnosed with active convulsive epilepsy. Epilepsy patients’ out-of-pocket, medical and non-medical costs and frequency of outpatient care visits during the previous 12-months were determined.

**Results:**

Within the last year, 132 (53 %) individuals reported consulting at a clinic, 162 (65 %) at a hospital and 34 (14 %) with traditional healers for epilepsy care. Sixty-seven percent of individuals reported previously consulting with both biomedical caregivers and traditional healers. Direct outpatient, median costs per visit varied significantly (*p* < 0.001) between hospital (2010 International dollar ($) 9.08; IQR: $6.41-$12.83) and clinic consultations ($1.74; IQR: $0-$5.58). Traditional healer fees per visit were found to cost $52.36 (IQR: $34.90-$87.26) per visit. Average annual outpatient, clinic and hospital out-of-pocket costs totaled $58.41. Traveling to and from and waiting to be seen by the caregiver at the hospital took significantly longer than at the clinic.

**Conclusions:**

Rural South Africans with epilepsy consult with both biomedical caregivers and traditional healers for both epilepsy and non-epilepsy care. Traditional healers were the most expensive mode of care, though utilized less often. While higher out-of-pocket costs were incurred at hospital visits, more people with ACE visited hospitals than clinics for epilepsy care. Promoting increased use and effective care at clinics and reducing travel and waiting times could substantially reduce the out-of-pocket costs of outpatient epilepsy care.

**Electronic supplementary material:**

The online version of this article (doi:10.1186/s12913-016-1460-0) contains supplementary material, which is available to authorized users.

## Background

Epilepsy is a common neurological disorder with 80 % of all cases found in low- and middle-income countries (LMICs) [1]. Ten million people live with epilepsy on the African continent alone [2]. Studies from LMICs have quantified the epidemiological burden of epilepsy - consistently showing higher incidence [3] and mortality [4] associated with epilepsy. However, few population-based studies have explored the utilization and cost of health care due to epilepsy in these countries, with no known studies from South Africa.

Due to its chronic, stigmatizing, and often debilitating nature, epilepsy carries significant economic costs in terms of both medical treatment and lost economic productivity [5]. Studies have found costs to vary by seizure type and frequency, temporal stage of the disorder, diagnostic and treatment tools available, and the frequency and type of health care services utilized by people with epilepsy [6]. A review of 22 cost-of-illness studies on epilepsy from both low- and middle-income and high-income countries found mean annual direct costs (costs related to seeking and receiving care for epilepsy) to range from 2006 International dollar ($) 40 to $4768, with costs substantially lower in LMICs, ranging from $40 to $384 [7]. Anti-epileptic drugs (AEDs) and hospital admissions are major drivers of direct costs associated with epilepsy treatment [7].

The few African studies that exist find that costs due to epilepsy can be substantial, with the majority of costs due to AEDs [7]. A population-based Burundian study found people with epilepsy to have significantly higher annual health care costs than those without any neurological condition, with those taking AEDs experiencing a 440 % increase [8]. A Nigerian study, exploring the cost of pediatric epilepsy at a tertiary health facility, found that more than half of all families with a household member with epilepsy spent more than 20 % of the family’s total income on their care [9].

### Health care utilization

Health care utilization is an important component of both costs and adherence, with the relationship between these factors often bidirectional and complex [10, 11]. Studies from high-income countries show that people with epilepsy utilize health care more often than people without epilepsy due to repeat drug prescriptions [12] and increased emergency room usage [13].

Existing studies in LMICs find a large epilepsy treatment gap - defined as those not on treatment or on inadequate treatment [14]. An analysis of 27 studies from LMICs found an overall treatment gap of 56 % [15]. This suggests that people do not utilize (or receive) the health care they need. Cost of treatment, unavailability of drugs, and inadequate healthcare manpower all contributed significantly to the observed treatment gap [15]. By understanding costs and health care utilization for people with epilepsy, interventions to remove barriers and improve access to care can be developed. In South Africa, where standard AEDs are provided free of charge, understanding drivers of out-of-pocket costs and time spent seeking health care may provide insights on how to reduce the treatment gap.

In the current study, we determined the health care utilization of people with active convulsive epilepsy, estimated the time spent seeking outpatient treatment and calculated direct out-of-pocket costs in a rural South African context. This is the first study of its kind from South Africa.

## Methods

### Study area and population

The Agincourt Health and Socio-Demographic Surveillance System (HDSS) site, located in the Agincourt health sub-district of the Bushbuckridge municipality, comprises 420 km^2^ of semi-arid, rural scrubland, 500 km northeast of Johannesburg, South Africa in Mpumalanga Province adjacent to Mozambique. Established as a research site in 1992, the Agincourt HDSS (http://www.agincourt.co.za) aims to inform health and development policy through evidence-based research and has generated socio-demographic data since its inception through annual census updates that capture vital statistics, including births, deaths, and in- and out-migrations.

The 2011 mid-year population was roughly 90,000 individuals in 16,000 households and 27 research villages [16]. The majority of the population speaks Xitsonga with one-third of the population of Mozambican origin.

Unemployment remains high in Agincourt with 36 % of the adult population (older than 18 years old) unemployed and currently looking for work [17]. The socio-economic status of the site has improved over the last 20 years, though as of 2011, 20 % of the population was still living in extreme poverty (personal communication, Mark Collinson). Government grants provide much needed financial support, with the majority of Agincourt households receiving at least one (personal communication, Mark Collinson).

Within the Agincourt site, a network of six, government primary health care (PHC) clinics offers free basic, outpatient health services during regular business hours. These services include mother and child care, including immunizations, family planning, testing and treatment for sexually transmitted infections, including HIV, minor trauma and routine care for those with chronic conditions, including epilepsy [18].

The six clinics refer patients to one larger government community health centre within the site. The health centre provides PHC services as well as 24-h acute care and facilities for 48-h patient observation [18]. Nurses run both clinics and health centres, though doctors do visit clinics and health centres.

A public-private partnership community health centre within the site provides HIV, tuberculosis and other chronic disease care and treatment. Clinicians staff this facility.

Referrals from primary health care facilities in the Agincourt research site are made to three government hospitals located 25-55 km from the site. Clinicians at the hospitals primarily diagnose epilepsy and initiate therapy before referring patients to the clinics or health centre for monthly AED collection [19]. People with epilepsy are expected to return to the hospital at 6-month intervals for review of seizure frequency and treatment.

### Case definition and case ascertainment

A 2008 cross-sectional survey sought to identify all individuals with active convulsive epilepsy (ACE) living within the Agincourt HDSS [20]. ACE was defined as having ≥2 unprovoked convulsive seizures occurring more than 24 h apart, with at least one seizure occurring in the year preceding the study or currently taking AEDs for epilepsy [20]. After undergoing a clinical history and neurological exam, 311 individuals living within the Agincourt HDSS were diagnosed with ACE by the study neurologist (CRN), yielding an adjusted ACE prevalence of 7.0 per 1000 individuals [20].

### Data collection

Between September 2010 and January 2011, trained fieldworkers attempted to visit each individual diagnosed with ACE and/or his/her caregiver. If consent was given, the respondent was asked questions regarding health care utilization over the previous 12-months. Questions on out-of-pocket costs associated with utilization of outpatient care for the treatment of ACE were asked regarding the last visit to the biomedical health care facility. The questionnaire was forward- and back- translated into the local language and piloted with local community members. The English version of the questionnaire is available as an Additional file (Additional file 1).

### Statistical analysis

The questionnaires were entered into a mySQL database (OracleCorp, Redwood Shores, CA, U.S.A) by a trained data typist and the data analyzed using Stata 13 (StataCorp, College Station, TX, U.S.A). Due to the non-normal distribution of health care and traditional healer visits and out-of-pocket costs (*p* < 0.0001 when performing the Shapiro-Wilk test of normalcy), we report results as median and interquartile range.

The annual cost of outpatient, out-of-pocket care was calculated by multiplying the average cost per visit by the average number of visits reported in the last 12-months.

The time traveling to a traditional healer was not collected in this study and only traditional healer fees are reported as results.

## Results

Household visits were made to all 311 individuals diagnosed with ACE in 2008; 20 were found to be deceased, 8 had permanently out-migrated from the study area, 9 refused to participate in the study and 24 were not available after visiting the household on at least 3 separate occasions. There was no significant age or sex variation between respondents and non-respondents. Of those alive and found, 250 individuals (97 %) agreed to take part in the study with a male-to-female ratio of 1.05. The median age was 29 (inter-quartile range: 25^th^ percentile- 75^th^ percentile (IQR): 18–44 years). Seven (14 %) of those older than 18 years were currently employed whilst 33 (50 %) of those younger than 18 years were currently in school. One hundred forty-eight individuals (59 %) were receiving an epilepsy-related, government disability grant of $188 per month, whilst 4 individuals (2 %) reported receiving smaller non-epilepsy-related grant amounts ranging from $44-176 (Table 1).

**Table 1:** Socio-demographic characteristics of study participants (n)

	n (%)
Age band	
0–5	9 (4)
6–12	27 (11)
13–18	31 (12)
19-28	54 (22)
29–49	85 (34)
50+	44 (17)
Sex	
Male	128 (51)
Female	122 (49)
Receiving government grant	
Yes	153 (61)
No	97 (29)
Socio-Economic Status^a^	
1st Quintile	31 (13)
2nd Quintile	65 (27)
3rd Quintile	50 (20)
4th Quintile	50 (20)
5th Quintile	49 (20)
Currently employed (if >18 years old)	
Yes	14 (8)
No	169 (92)
Currently in school (if <18 years old)	
Yes	33 (49)
No	34 (51)

^a^derived from 2009 Agincourt SES module; 5 missing data

### General health care utilization in people with epilepsy

Two hundred thirty-nine (96 %) individuals reported receiving non-epilepsy related health care when they last needed it, which for 154 individuals (62 %) was in the previous 30-days. For 231 (91 %) individuals, it was in the last year. Four individuals (1 %) reported being denied care and one participant cited inadequate levels of biomedical caregiver’s skill as reason for not receiving the necessary health care. Six individuals did not report the reason for not receiving care when they last needed it.

One hundred eighty-four (74 %) had ever sought general care from both a biomedical facility (hospital, health centre, or clinic) and a traditional healer, whilst 63 individuals (25 %) only sought general care from a biomedical facility and 1 person sought general care only from a traditional healer.

### Epilepsy-related health care utilization

Within the last year, 132 people with epilepsy (53 %) report consulting for epilepsy at a clinic, 162 (65 %) at a hospital and 34 (14 %) with traditional healers. One hundred sixty-eight individuals (67 %) reported previously seeking epilepsy care from both a biomedical facility and a traditional healer, whilst 61 individuals (24 %) have sought care from only biomedical facilities. Eleven individuals (4 %) reported only seeking care for epilepsy from traditional healers, whilst 10 individuals sought no care- neither from a biomedical facility nor a traditional healer. People with epilepsy sought general care at the government clinic more frequently than epilepsy-related care (94 % versus 76 %), whilst utilizing hospital care at similar levels for both epilepsy and general care (91 % versus 86 %) (Table 2). The average number of hospital visits in the previous 12-months was 4.06 (standard deviation (SD): 4.75) and average number of clinic visits was 6.5 (SD: 5.75).

**Table 2:** Lifetime utilization of care by people with epilepsy & median number of visits in previous year, Agincourt 2010–11 (*n* = 250)

	Ever sought attention for General care (%)	Median visits in previous 12-months (IQR)	Ever sought attention for epilepsy-related care (%)	Median visits in previous 12-months (IQR)
Government Clinic	235 (94)	1 (0–5)	189 (76)	10 (0–11)
Government Hospital	228 (91)	0 (0–1)	216 (86)	2 (0–10)
Traditional Healer	185 (74)	0 (0–0)	179 (73)	0 (0–0)

### Costs of biomedical health care utilization

The median cost per clinic visit was found to be $1.74 (IQR: $0-$4.80) (mean: 2.74; SD 3.74), whilst the median cost per outpatient hospital visit was found to be $9.08 (IQR: $6.11-$12.91) (mean: 9.73; SD 5.34) – a statistically significant difference (*p* < 0.001). The costs for epilepsy care at clinic and hopsital can be found in Table 3. The average annual cost for out-of-pocket, outpatient biomedical care at a clinic or hospital was found to be $58.41.

**Table 3:** Median direct costs for epilepsy care at clinic and hospital, Agincourt 2010-11

	Clinic	Hospital
Direct Medical Costs (per visit)		
Epilepsy Medication	0 (0–0)	0 (0–0)
Opening file	$ 3.49 (3.49–5.24)	$ 3.49 (3.49–3.49)
Direct Non-Medical Costs (per visit)		
Transportation	$ 2.79 (1.74–4.89)	$ 5.41 (4.19–7.68)
Food/drink purchased due to visit	$ 3.49 (1.74–5.24)	$ 4.36 (3.49–6.98)

Median (IQR)

Expressed in 2010 International dollars $

The median travel and waiting time at the hospital was twice that of the clinic (120 min/60 min and 240 min/120 min, respectively), whilst the time spent with a health professional was the same (10 min) (Table 4). The majority of respondents walked to the clinic but took a public taxi to the hospital. During their last visit to the hospital, 30 % of respondents saw a physician and 70 % saw a nurse. During their last visit to the clinic, 11 % saw a physician and 89 % saw a nurse.

**Table 4:** Time associated with seeking epilepsy care (traveling, waiting and consulting), Agincourt 2010–11

Travel Time	Median Time (in minutes)	IQR	Mode of Transport
to Clinic	60	40–90	62 % walked, 38 % public taxi
to Hospital	120	120–240	92 % public taxi, 4 % walked, 3 % hired car, 1 % personal car
Waiting Time			
to Clinic	120	10–420	
to Hospital	240	30–600	
Time with Health Professional			Health Professional Seen
to Clinic	10	9–15	11 % physician, 89 % nurse
to Hospital	10	10–15	30 % physician, 70 % nurse
Time Totals			
Clinic	220*	140–310	
Hospital	490*	320–610	

*p < 0.0001

### Costs of traditional healer utilization

During the last visit to a traditional healer 160 individuals (87 %) had to pay for services, with payment in all cases being cash. The median cost for a traditional healer’s services was $52.36 ($34.90-$87.26). One individual reported only having to pay the traditional healer if s/he was cured of epilepsy.

## Discussion

We found that the majority of people with ACE (91 %) needed non-epilepsy care in the previous year, which was similar to the 87 % percent in those greater than 50 years old who reported needing care within the previous year in a separate study within the same site [21]. Additionally, 53 % of people with ACE sought care for epilepsy from a clinic and 65 % from a hospital in the previous year. One limitation of the current study is that we have no indication as to whether the utilization of outpatient, biomedical care was routine or the result of an epilepsy-related emergency. Future investigation is warranted to address this.

Findings from high-income countries suggest that people with epilepsy utilize health care more often than individuals with other chronic conditions, including asthma, diabetes and migraines [12, 13, 22]. People with epilepsy in the Agincourt sub-district are required to visit the clinic once per month to collect AEDs. This requirement may result in the higher clinic utilization levels found in people with epilepsy when compared to those greater than 50 years old with other chronic conditions in the area [23]. Further work to ascertain the reasons for increased health care utilization and to determine whether people with epilepsy benefit from the care they seek in this rural southern African setting is also warranted.

People with epilepsy were more likely to utilize public clinics for non-epilepsy care, whilst more likely to utilize public hospitals for epilepsy care. The frequency of the visits to the clinic, for both general care and epilepsy-related care, was greater than the frequency of hospital visits. The reason for more people seeking care at the hospital rather than the clinic is unclear. Perceived inadequacy of skilled manpower has been cited as a cause for people not seeking care in a number of studies from LMICs [24–26], and the perception of ‘more adequate’ manpower at the hospital (even though only 30 % of patients saw a doctor at the hospital, compared to 11 % at the clinic) could potentially explain a patient’s preference to attend hospital for epilepsy care.

The non-availability of AEDs has also been shown to affect care-seeking patterns [15] and is another possible reason that some people with epilepsy chose to seek care at the hospital rather than the clinic. Hospitals, with larger catchment areas, are likely to have a more continuous supply of medication. In this study, 10 % of patients reported that drugs are not always available and 22 % of patients reported that the lack of drugs discouraged them from utilizing the health facility. While this study didn’t explore the percentage of stock-outs at each facility, this research does suggest that improving drug supply could encourage better PHC facility attendance.

### Traditional healer utilization

We found disclosed lifetime traditional healer usage to be high in people with epilepsy for both epilepsy- and non-epilepsy-related care (73 % and 74 %, respectively), although the frequency of visits for both general and epilepsy-related care was substantially less than visits to both biomedical facilities within the 12 months preceding this study. A national South African survey found similar patterns, though lower utilization levels, and suggests that poorer, rural, black populations utilize traditional healers more than urban, non-black populations [27].

Utilization of traditional healers within the previous 12 months was low when compared to those who reported ever seeking traditional healer care for epilepsy. It may be that traditional healer care was sought during the initial onset of seizures and care discontinued as the seizures continued and traditional treatment proved ineffective. Still, nearly 15 % of people with epilepsy reported at least one visit to the traditional healer for epilepsy care within the previous 12-months.

Our research concurs with findings that suggest the costs associated with traditional healers are substantially higher than costs of seeking care at biomedical facilities [28]. The traditional healers’ costs we present only include traditional healers’ fees and do not include time and money spent traveling to and waiting to be seen by a traditional healer, which would likely increase the total costs. Our results likely represent the lower bound of costs associated with traditional healer costs. The higher cost per visit may be financially prohibitive and result in the lower frequency of utilization. Furthermore, the aggregate cost of traditional healers, taking into account the lower frequency but higher per visit cost, may be similar to the cost of biomedical facilities, which are utilized more often, but cost, per visit, substantially less.

Understanding determinants of traditional healer utilization in this context is warranted as the cultural understanding of epilepsy has been shown to be an important determinant in care-seeking behavior [29]. Identifying ways of working with traditional healers to reduce the epilepsy treatment gap, however, may be more productive than attempting to change individual’s behavior.

### Costs associated with epilepsy treatment

Seeking care at the hospital took significantly longer and cost significantly more- more than twice the cost of seeking care at the clinic. As costs (including lost time) associated with seeking epilepsy treatment have commonly been found to be associated with the epilepsy treatment gap [15], increasing the utilization of care at clinics by strengthening epilepsy care delivery will likely reduce costs and potentially improve the treatment gap. Furthermore, reducing transportation cost could result in an overall reduction in out-of-pocket costs given that transportation costs have been highlighted as a significant cost in previous work in the area [28].

The overall annual out-of-pocket costs for out-patient epilepsy care at biomedical facilities was found to be $58.41, on average, yet this only represents a portion of the total cost of epilepsy care in rural South Africa.

### AED costs

Patients’ AED costs are likely to be lower in South Africa than other LMICs due to the free availability of AEDs at health facilities. No patient reported paying for AEDs in this study. In contrast, two other African studies found AEDs to contribute 10 % [8] and 22 % [9] of the total direct epilepsy treatment-related costs. One study from rural Kenya found that people with epilepsy who had to pay for AEDs were less likely to seek care [30], suggesting that reducing or removing the cost of AEDs might improve accessibility and consequently adherence [31]. Future work exploring adherence patterns in South Africa, where AEDS are free, is warranted and could be a natural experiment to establish whether free availability of AEDs to patients truly does improve adherence.

### Limitations

While this study presents previously unavailable information on utilization patterns and out-of-pocket costs for people with ACE in rural South Africa, there are a number of potential limitations.

We only examined people with ACE in this study. We recognize that this cohort does not represent all epilepsies and limits the interpretation of the results of this study to people with ACE only. However, convulsive epilepsies are often associated with the largest morbidity, stigma and mortality [32] and potentially pose a greater burden to individuals and families compared to non-convulsive epilepsies.

This study did not measure indirect costs associated with ACE, whilst a number of studies have found that this cost can be significant- ranging from 12 % to 85 % of the total annual costs due to epilepsy [7]. As such, this study likely underestimates the total cost of ACE. This study also only explored out-of-pocket, outpatient costs associated with ACE and did not include costs associated with hospital admissions or epilepsy-related surgeries (in-patient costs). Studies from Italy have found in-patient costs to be significant, especially in new-onset epilepsy [33, 34]. This study only examined ACE, and outpatient treatment patterns and costs for other types of epilepsy may vary; however, further studies are needed to verify this.

We chose an out-of-pocket costing approach for this study. In countries, such as South Africa, with high levels of unemployment and substantial inequality, using the GDP per capita as a proxy for productivity may not represent the actual productivity loss due to epilepsy and, some argue, fails to account for the value of human life [35, 36]. As such, a wider, societal costing approach was not adopted in this study. Further development of methodologies to allow for the accurate calculation of productivity costs in areas of high unemployment where formal sector employment is scarce is needed.

All costs and utilization analyzed in this study were self-reported. Recall bias or intentional bias in reporting may result in misreporting of costs and utilization associated with seeking epilepsy care. Every effort, through careful probing by well-trained fieldworkers, was made to ensure the collection of accurate data.

Finally, this study only explored out-of-pocket costs of epilepsy to the patient. Given the free availability of AEDs in South Africa and the subsidized direct, medical health care costs within the public healthcare system, epilepsy-related treatment costs to the health care system are potentially greater than in other African countries.

## Conclusions

Out-of-pocket, direct, outpatient costs are likely to contribute only a portion of the total economic burden of epilepsy, with further research needed to determine in-patient care costs as well as indirect costs attributable to epilepsy in rural South Africa. However, this research suggests that rural South African patients spend more time and money seeking epilepsy care from a hospital than from a clinic and readily seek both biomedical and traditional healthcare. Interventions aimed at strengthening epilepsy care at the primary care clinic, including regular drug supply chains, and reducing the amount of time spent traveling to and waiting to be seen at biomedical facilities could substantially reduce the out-of-pocket costs experienced by people with active convulsive epilepsy.

## Abbreviations

$, International dollar; ACE, Active convulsive epilepsy; AEDs, Anti-epileptic drugs; HDSS, Health and demographic surveillance system; IQR, interquartile range; LMICs, Low- and middle- income countries; PHC, primary health care

## Additional file

Additional file 1: Questionnaire used for data collection, Agincourt 2010. (DOCX 153 kb)
