# Supplementary material for: Health care utilization and outpatient, out-of-pocket costs for active convulsive epilepsy in rural northeastern South Africa: a cross-sectional Survey
Source: BMC Health Serv Res. 2016 Jun 28;16:208. doi: 10.1186/s12913-016-1460-0 (PMC4924265; doi:10.1186/s12913-016-1460-0)
Supplement: Additional file 1: — Questionnaire used for data collection, Agincourt 2010. (DOCX 153 kb) [file 12913_2016_1460_MOESM1_ESM.docx]

|  | Agincourt DSS No. (PID) | | |  | | | | | | | | | | | | | |  | | | | | | | | | | | | | |
| --- | --- | --- | --- | --- | --- | --- | --- | --- | --- | --- | --- | --- | --- | --- | --- | --- | --- | --- | --- | --- | --- | --- | --- | --- | --- | --- | --- | --- | --- | --- | --- |
|  | Location | | | \| Village: \|  \| \|  \|  \| \| --- \| --- \| \| 1 \| 1 \| \| \| --- \| --- \| --- \| --- \| --- \| --- \| --- \| \|  \|  \|  \| \| Dwelling: \|  \|  \| | | | | | | | | | | | | | | | | | | | | | | | | | | | |
|  | Name and Surname | | | \|  \|  \| \| --- \| --- \| | | | | | | | | | | | | | | | | | | | | | | | | | | | |
|  | Gender | |  | | | | | | | | | | | **1** = Male  **2** = Female | | | | | | | | |  | | | | | | |  | |
|  | Date of Birth | |  | | | | | |  | | | | | | | | | |  | | | | | | | | | | | | |
|  | Do you know that you can receive a disability grant for epilepsy? | | | | | | | | | | | | | | | | **1** = Yes **2** = No | | | | | | | | |  | | |  | | |
|  | Do you receive a disability grant for epilepsy? | | | | | | | | | | | | | | | | **1** = Yes **2** = No | | | | | | | | |  | | |  | | |
|  | How much do you receive from the disability grant? (in ZAR) | | | | | | | | | | | | | | | |  | | | | | | | | |  | | | | | |
|  | Do you seek medical attention from a clinic? | | | | | | | | | | | | | | | | **1** = Yes **2** = No | | | | | | | | |  | | |  | | |
|  | How many times have you sought medical attention at a clinic in the last year? | | | | | | | | | | | | | | | | | | | | | | | | |  | | |  | | |
|  | Do you seek epilepsy treatment from a clinic? | | | | | | | | | | | | | | | | **1** = Yes **2** = No | | | | | | | | |  | | |  | | |
|  | How many times have you sought epilepsy treatment at a clinic in the last year? | | | | | | | | | | | | | | | | | | | | | | | | |  | | |  | | |
|  | Do you seek medical attention from a hospital? | | | | | | | | | | | | | | | | **1** = Yes **2** = No | | | | | | | | |  | | |  | | |
|  | How many times have you sought medical attention at a hospital in the last year? | | | | | | | | | | | | | | | | | | | | | | | | |  | | |  | | |
|  | Do you seek epilepsy treatment from a hospital? | | | | | | | | | | | | | | | | **1** = Yes **2** = No | | | | | | | | |  | | |  | | |
|  | How many times have you sought epilepsy treatment at a hospital in the last year? | | | | | | | | | | | | | | | | | | | | | | | | |  | | |  | | |
|  | Do you seek medical attention from a traditional healer? | | | | | | | | | | | | | | | | **1** = Yes **2** = No | | | | | | |  | | | | |  | | |
|  | How many times have you sought medical attention from a traditional healer in the last year? | | | | | | | | | | | | | | | | | | | | | | |  | | | | |  | | |
|  | Do you seek epilepsy treatment from a traditional healer? | | | | | | | | | | | | | | | | **1** = Yes **2** = No | | | | | | |  | | | | |  | | |
|  | How many times have you sought epilepsy treatment from a traditional healer in the last year? | | | | | | | | | | | | | | | | | | | | | | |  | | | | |  | | |
|  | During your last visit to the traditional healer did you have to pay/give the traditional healer anything? | | | | | | | | | | **1** = Yes **2** = No | | | | | | | | | | | | |  | | | | |  | | |
|  | What did you pay/give the traditional healer? | | | | | | | | | | **1** = Money  **2** = Meat (chicken)  **3** = Grown Food (peanuts, mealies)  **4** = Other | | | | | | | | | | | | |  | | | | |  | | |
|  | How much money? (in ZAR) | | | | | | | | | | | | | | | | |  | | | | | |  | | | | | | | |
|  | Other | | | | | |  | | | | | | | | | | | | | | | | | | | | | | | | |
|  | When was the last time you needed healthcare? | | | | | | | | | | | | | | | |  | | | | | | | | | | | | | | |
|  | The last time you needed healthcare did you get health care? | | | | | | | | | | | | | | | | **1** = Yes  **2** = No | | | | | | | | | |  | |  | | |
|  | Which reason(s) best explain why you did not get health care? **Select all that apply** | | | | | | | | | | | | | | | | | | | | | | | | | |  | | | |  |
|  | **1** =(Could not afford the cost of the visit)  **2** =(No transport available)  **3** =(Could not afford the cost of transport )  **4** =(Treated poorly during a previous visit)  **5** =(Could not take time off of work; busy)  **6** =(Drugs or equipment seemingly inadequate)  **7 =** (Health provider’s skills seemingly inadequate)  **8** =(Did not know where to go)  **9** =(Tried but was denied health care)  **10** =(Did not think you were sick enough) | | | | | | | | | | | | | | | | | | | | | | | | | |  | | | |  |
|  |  |  |  |  |  |  |  |  |  |  |  |  |  |  |  |  |  |  |  |  |  |  |  |  |  |  |  | | | |  |
| **To the fieldworker:** Please ask the respondent to answer the following questions based on their most recent visit to a clinic/hospital for epilepsy consultation or treatment. | | | | | | | | | | | | | | | | | | | | | | | | | | | | | | | |
|  | At which clinic or hospital did you most recently seek treatment for epilepsy? | | | | | \| 27 \| \|  \| \| --- \| \| \| --- \| --- \| --- \| | | | | | | | | | | | | | | | | | | | | | | | | | |
|  | How did you travel to the clinic or hospital? | | | | | | | **1** = Walked **2** = Public Transport (taxi,bus)  **3** = Personal vehicle (car, motorbike)  **4** = Bicycle  **5** = Other | | | | | | | | | | | | | | | |  | | | | |  | | |
|  | Other (specify) | | | |  | | | | | | | | | | | | | | | | | | | | | | | | | | |
|  | How long did it take you to reach the clinic or hospital? (in minutes) | | | | | | | | | | | | | | | | | | | | |  | | | | |  | | | | |
|  | How much money did you spend on transport to and from the clinic/hospital? (in ZAR) | | | | | | | | | | | | | | | | | | | | |  | | | | |  | | | | |
|  | Other (specify) | | | |  | | | | | | | | | | | | | | | | | | | | | | | | | | |
|  | During your most recent visit, how long did you wait to be seen at the clinic or hospital? (in minutes) | | | | | | | | | | | | | | | | | | | | |  | | |  | | | | | | |
|  | What type of health care provider did you see at the clinic or hospital? | | | | | | | | | | | | | | | **1** = Doctor **2** = Nurse  **3** = Other | | | | | | | | |  | | | |  | | |
|  | Other (specify) | | | |  | | | | | | | | | | | | | | | | | | | | | | | | | | |
|  | How long did you spend with the health care provider at the clinic or hospital? (in minutes) | | | | | | | | | | | | | | | | | | | | |  | | | |  | | | | | |
|  | Did you receive medication while you were at the clinic or hospital? | | | | | | | | | | | | | | | | | | | **1** = Yes **2** = No | | | | | |  | | |  | | |
|  | Were your epilepsy drugs available at the clinic or hospital? | | | | | | | | | | | | | | | | | | | **1** = Yes **2** = No | | | | | |  | | |  | | |
|  | How much money did you spend on medication? (in ZAR) | | | | | | | | | | | | | | | | | | |  | | | | | |  | | | | | |
|  | Did you spend other money at the clinic or hospital? | | | | | | | | | | | | | | | | | | | | **1** = Yes **2** = No | | | | |  | | |  | | |
|  | What did you spend money on? How much?  *(Mark all appropriate circles and fill in adjacent figure in ZAR)* | | | | | | | | |  | | Food and Drink | | | | | | | | | | ⭘ | | | | | |  | | | |
|  |  |  |  |  |  |  |  |  |  |  | | Opening a file | | | | | | | | | | ⭘ | | | | | |  | | | |
|  |  |  |  |  |  |  |  |  |  |  | | Accommodation | | | | | | | | | | ⭘ | | | | | |  | | | |
|  |  |  |  |  |  |  |  |  |  |  | | Other | | | | | | | | | | ⭘ | | | | | |  | | | |
|  | Other (specify) | | | | |  | | | | | | | | | | | | | | | | | | | | | | | | | |
|  | | Are epilepsy drugs always available? | | | | | | | | | | | | | **1** = Yes **2** = No | | | | | | | | | | | |  | |  | | |
|  | | How many times in the last 12 months have epilepsy drugs not been available? | | | | | | | | | | | | | | | | | | | | | | | | |  | |  | | |
|  | | Does lack of drugs discourage you from going to the clinic/hospital? | | | | | | | | | | | | | **1** = Always **2** = Sometimes  **3** = Never | | | | | | | | | | | |  | |  | | |
|  | | How likely are you return for your next visit if drugs were not available during your previous visit? | | | | | | | | | | | | | **1** = More likely **2** = The same  **3** = Less likely | | | | | | | | | | | |  | |  | | |
|  | Do you have a job? | | | | | | | | |  | | | **1** = Yes **2** = No | | | | | | | | | | | |  | | | |  | | |
|  | Do you go to school? | | | | | | | | |  | | | **1** = Yes **2** = No | | | | | | | | | | | |  | | | |  | | |
